# Unravelling Linkages between Plant Community Composition and the Pathogen-Suppressive Potential of Soils

**DOI:** 10.1038/srep23584

**Published:** 2016-03-29

**Authors:** Ellen Latz, Nico Eisenhauer, Björn Christian Rall, Stefan Scheu, Alexandre Jousset

**Affiliations:** 1 J.F. Blumenbach Institute of Zoology and Anthropology, Georg August University Göttingen, Berliner Straße 28,37073 Göttingen, Germany; 2 German Centre for Integrative Biodiversity Research (iDiv) Halle-Jena-Leipzig, Deutscher Platz 5e, 04103 Leipzig, Germany; 3 Institute of Ecology, Friedrich Schiller University Jena, Dornburger Straße 159, 07743 Jena, Germany; 4 Institute of Biology, Leipzig University, Johannisallee 21, 04103 Leipzig, Germany; 5 Institute for Environmental Biology, Ecology and Biodiversity, University of Utrecht, Padualaan 8, 3584CH Utrecht, The Netherlands

## Abstract

Plant diseases cause dramatic yield losses worldwide. Current disease control practices can be deleterious for the environment and human health, calling for alternative and sustainable management regimes. Soils harbour microorganisms that can efficiently suppress pathogens. Uncovering mediators driving their functioning in the field still remains challenging, but represents an essential step in order to develop strategies for increased soil health. We set up plant communities of varying richness to experimentally test the potential of soils differing in plant community history to suppress the pathogen *Rhizoctonia solani*. The results indicate that plant communities shape soil-disease suppression via changes in abiotic soil properties and the abundance of bacterial groups including species of the genera *Actinomyces, Bacillus* and *Pseudomonas*. Further, the results suggest that pairwise interactions between specific plant species strongly affect soil suppressiveness. Using structural equation modelling, we provide a pathway orientated framework showing how the complex interactions between plants, soil and microorganisms jointly shape soil suppressiveness. Our results stress the importance of plant community composition as a determinant of soil functioning, such as the disease suppressive potential of soils.

Soil-borne plant pathogens cause important crop yield losses all over the world^1^,^2^. Some especially aggressive pathogens, such as *Fusarium, Pythium* and *Rhizoctonia,* can cause losses of up to 20–35%^3^,^4^. Current control methods are based on pesticide application, which are highly polluting and provide only partial protection^1^,^5^.

Pathogen Some soils naturally suppress diseases, an effect linked to distinct biological mechanisms^1^. Bacteria of the genera *Pseudomonas, Actinomyces* and *Bacillus* are particularly important for the suppressiveness of soils^1^,^6^, and their targeted application is offering the opportunity for environmentally friendly control of plant diseases^1^,^5^. However, despite of extensive research on the molecular mechanisms involved in disease suppression by bacteria^5^,^6^,^7^,^8^, there still is a lack of knowledge on drivers affecting their survival and functioning in the soil^9^,^10^.

Plant diversity affects a variety of ecosystem functions and services^11^,^12^, and drives the structure and antifungal activity of bacterial communities antagonistic to plant pathogens^13^,^14^,^15^,^16^. Generally, effects of plant diversity on microbial communities are suggested to be due to increased plant productivity, accompanied by an increased quantity of resources exudated by plant roots^17^,^18^. In contrast to overall microbial community functioning and productivity, specific ecosystem functions, such as the performance of plant-pathogenic as well as pathogen-antagonistic microorganisms might be driven by resource quality creating certain rhizosphere conditions^19^,^20^. For instance, in the presence of competitors plants invest more in root exudates providing certain functions, such as nutrient foraging, facilitative plant-plant communication and allelopathy, which likely affect the rhizosphere microbial community^19^. Further, plant species specific root exudates impact not only the nutritional status in the rhizosphere, but also important microbial drivers such as pH^21^,^22^. In addition to abiotic rhizosphere properties shaped by root exudates, soil-moisture is a component that varies with plant communities and shapes soil microbial communities^22^. However, factors responsible for plant community effects on rhizosphere microbial communities and their functioning are little studied^23^.

Here, we attempt to reveal and disentangle pathways linking plant diversity to disease suppression. We hypothesised that abiotic and biotic properties of the rhizosphere jointly shape the pathogen suppressive potential of soils. We assumed changes in the rhizosphere environment (root biomass, soil C/N ratio as a measure of nutritional status, pH and soil moisture) to vary with plant community composition and in turn affect the abundance and composition of biocontrol bacterial communities, thereby altering pathogen suppression (see Table 1 for a detailed description on the hypotheses). We set up a series of plant communities comprising grasses and legumes, two functional groups linked to disease suppression in previous experiments^14^,^15^. We used a structural equation modelling approach to assess 1) whether plant community composition affects soil abiotic and biotic parameters and 2) if these alterations predict suppression of the pathogen *Rhizoctonia solani* evaluated in a subsequent bioassay. Finally, we assessed whether plant-plant interactions drive soil disease suppression beyond species richness or functional group effects (Fig. 1).

## Results

### Structural equation model (SEM)

**Figure 2:**
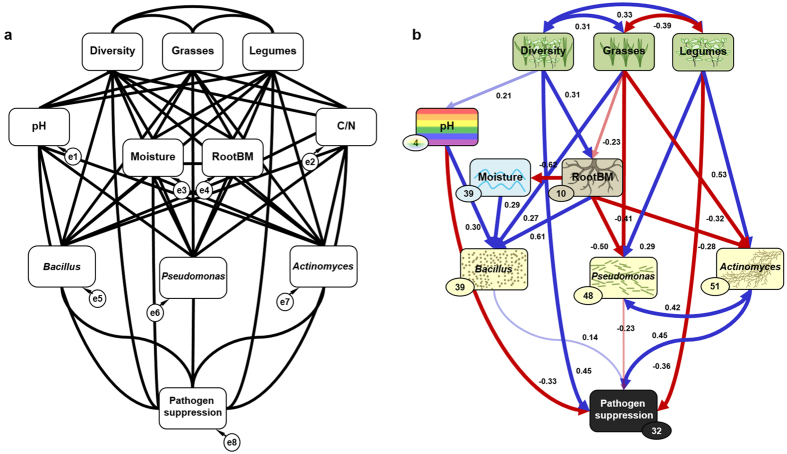
Structural equation model. The initial model (**a**) and the final model (**b**) with direct and indirect (through changes in soil pH, root biomass, soil moisture, and bacterial abundances) effects of plant community composition on pathogen suppression. Exogenous variables (plant diversity and functional group affiliation) are given on top, endogenous variables below. The data did not significantly deviate from the respective models (see main text for model fits). Single-headed arrows represent causal relationships and double-headed arrows indicate undirected correlations. Numbers on arrows give standardized path coefficients. Blue arrows indicate positive and red negative relationships; bold arrows indicate significant (P ≤ 0.05), medium size arrows indicate marginally significant (P ≤ 0.1), and thin arrows non-significant (P > 0.1) estimates. Circles indicate error terms (e1–e8). Numbers close to endogenous variables indicate the variance explained by the model (R^2^; percent).

Structural equation modelling revealed pathogen suppression to be affected by multiple pathways that are shaped by plant community composition. The initial model (χ^2^_11_ = 65.30; p < 0.001; Fig. 2a; Supplementary Table S1) could be improved by (i) using linear models to separately predefine each endogenous variable and its main dependencies to set up a second SEM (AICc = −1640.10; χ^2^_22_ = 27.64; p = 0.130), (ii) checking model modification indices, and (iii) removing non-significant pathways (AICc = −1649.73; χ^2^_21_ = 17.22; p = 0.698). The final model explained 32% of the variance in pathogen suppression (Fig. 2b; Supplementary Table S1).

Plant diversity increased root biomass production as well as soil pH, thereby indirectly increasing the abundance of *Bacillus* and subsequently pathogen suppression (although the effect being weak). In addition, the abundance of *Bacillus* was increased in the presence of grasses, while grasses slightly decreased root biomass. Root biomass, in turn, indirectly decreased the abundance of *Bacillus* via decreasing soil moisture. Further, an increase in soil pH was associated by a decrease in pathogen suppression. Despite the identified indirect pathways, a direct positive effect of plant diversity on pathogen suppression remained in the final model. The presence of legumes increased the abundances of *Pseudomonas* and *Actinomyces*. Furthermore, the presence of legumes directly decreased pathogen suppression. While being positively correlated, the abundance of *Pseudomonas* and *Actinomyces* decreased with root biomass and in the presence of grasses. Pathogen suppression increased with increasing abundance of *Actinomyces*, whereas it marginally decreased with increasing abundance of *Pseudomonas* (Fig. 2b, Supplementary Table S1).

### Plant-plant interaction analyses

**Figure 3:**
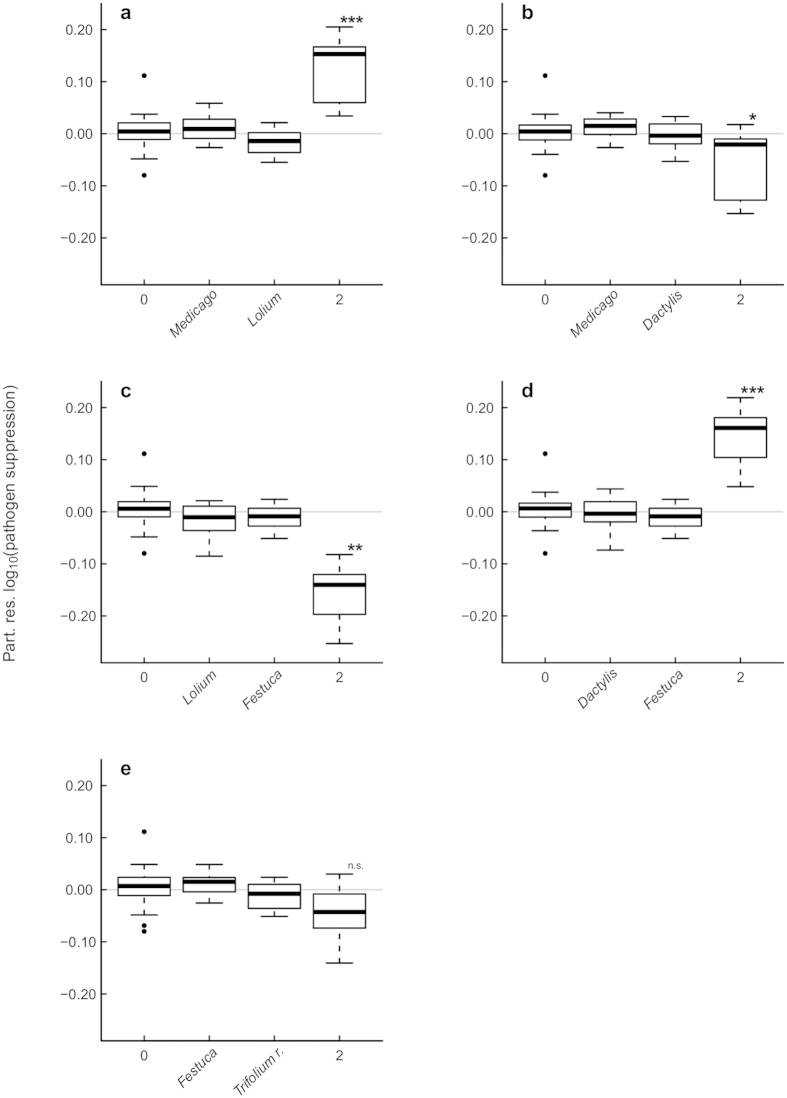
Partial residuals of log_10_-transformed pathogen suppression. (**a**) *Medicago* and *Lolium*, (**b**) *Medicago* and *Dactylis*, (**c**) *Lolium* and *Festuca*, (**d**) *Dactylis* and *Festuca*, (**e**) *Festuca* and *Trifolium r.* First box per graph indicates both plant species being absent “0”; the second and third box indicate named plant species being present and the other being absent; the fourth box indicates both plant species being present “2” (left to right). Interaction effects were tested against zero (two-tailed t-test). Asterisks denote the level of significance: *P ≤ 0.05, **P ≤ 0.01, ***P < 0.001.

**Figure 4:**
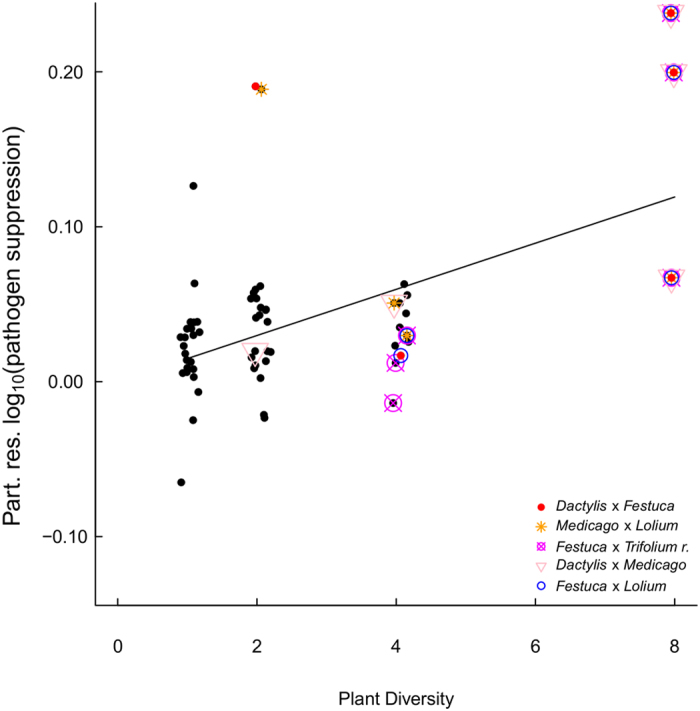
Partial residuals of log_10_-transformed pathogen suppression as affected by plant diversity. Displayed data is according to the most parsimonious model of the interaction analyses.

Analysing the residuals of the final SEM indicated that beyond plant diversity and legume presence, specific plant-plant interactions play an important role in influencing the pathogen suppressive potential of soil (Fig. 3). Here, the most parsimonious model included the species pairs *Medicago*-*Lolium* and *Dactylis*-*Festuca* that increased pathogen suppression (Fig. 3a,d), and *Medicago*-*Dactylis* and *Lolium*-*Festuca* that decreased pathogen suppression (Fig. 3b,c). Further, the species pair *Festuca*-*Trifolium r.* remained in the most parsimonious model and slightly decreased pathogen suppression (Fig. 3e). Interestingly, the positive effects of *Medicago*-*Lolium* and *Dactylis*-*Festuca* were most pronounced at plant diversity level 2, whereas the negative effects of the species pairs *Festuca*-*Lolium* and *Festuca-Trifolium r.* were most pronounced at diversity level 4 (Fig. 4). Interactions explained additional 32% of the remaining variance (after fitting the SEM) in pathogen suppression, resulting in approximately 54% explained variance in total.

### Additional analyses

Interestingly, when investigating whether the plant diversity effect was due to the presence of single species (sampling-effect)^24^,^25^ by fitting the presence of *Bromus, Dactylis, Festuca, Lolium, Lotus, Medicago, Trifolium p.* and *Trifolium r.* separately in a linear regression and fitting the residuals of the respective analyses against plant diversity^15^, the plant diversity effect remained significant only when fitted after the presence of some legume species (Supplementary Table S2). Further, the significance of the diversity effect disappeared when fitted after both the number of legume and the number of grass species (Supplementary Table S2), suggesting that single species were of minor importance.

## Discussion

A main challenge of sustainable agriculture is to enhance productivity of crop and grassland systems while minimizing inputs of pesticides and fertilizers. Fostering microbial communities that inhibit plant pathogens represent a promising approach to achieve this goal^10^,^26^. Microbial growth can be directly driven by abiotic soil factors, such as pH and humidity^27^. Further, soil microbial communities may inhibit pathogens by competing for space or nutrients or by inhibiting pathogens by the production of antibiotics^5^. Both abiotic and biotic parameters can be driven by plant community composition^8^,^10^,^28^. However, so far research neglected the complex linkages taking place in the rhizosphere when determining the suppressive potential of soils. In the present study we provide a pathway orientated framework showing how the complex interactions between plants, soil and microorganisms jointly shape soil suppressiveness.

In the present study, pathogen suppression was indeed influenced by a complex set of abiotic as well as biotic rhizosphere properties that are linked, directly or indirectly, to plant community composition. Plant community composition affected pH and the abundance of *Actinomyces*, which were both positively related to the suppression against *R. solani*. Further, certain interactions between plant species explained a large proportion of pathogen suppression in addition to the presence of plant functional groups and plant diversity *per se*. This suggests that plant community effects on soil abiotic and biotic properties alter microbial consortia in the rhizosphere and interactions therein, which need to be taken into account for predicting and manipulating the disease suppressive potential of soils. The results represent an important step forward in understanding the complexity of linkages between plant community composition and plant disease suppression.

Generally, our results underline the importance of plant diversity as an important determinant of soil suppressiveness. This is in line with studies showing that (1) soil suppressiveness rapidly vanishes during the conversion of grasslands to monocultures, which was ascribed to the decline in microorganisms being able to suppress diseases^13^, (2) soils from species-rich grasslands host high abundances of bacteria associated with pathogen suppression^14^, and (3) species-rich plant communities support high levels of the expression of genes that are associated with antifungal activity^15^. However, soil suppressiveness against pathogens (including *R. solani*) may also be fostered in continued monocultures showing disease symptoms^1^. In our control treatments (not inoculated with *R. solani*) only a small proportion of sugar beet seedlings got infected by pathogens (only in 9 controls we observed symptoms of disease; see http://idata.idiv.de/DDM/Data/ShowData/61 for details). Further, the initial infection in the treatments where controls showed infections occurred not significantly later than in the treatments that showed no infected controls (F-test; p = 0.285). Therefore, we suppose that the induction of suppressiveness due to indigenous *R. solani* occurance played a minor role in our experiment.

The SEM approach revealed that part of the plant diversity effect on the community structure and functioning of biocontrol bacteria was mediated by increased root biomass and soil pH. This is in line with a recent field study on experimental grassland, showing that plant diversity increases root biomass and soil pH, and thereby microbial biomass in soil^29^.

In addition to plant species richness, the presence of the functional groups grasses and legumes also predicted suppressiveness and again the effects were partly mediated by changes in root biomass and microbial communities. Generally, each plant functional group selected for different biocontrol bacteria. Grasses increased the abundance of *Bacillus*, and decreased the abundance of *Pseudomonas* and *Actinomyces*. Interestingly, as indicated by our SEM approach, the presence of grasses also indirectly increased the abundance of *Pseudomonas* and *Actinomyces* via decreasing root biomass, whereas legumes directly increased the abundance of *Pseudomonas* and *Actinomyces.* This contrasts with earlier studies, where legumes detrimentally and grasses beneficially affected the abundance of pseudomonads carrying genes linked to the production of antifungal compounds^14^. However, effects of legumes and grasses seem to be species specific^15^, and functional group effects on biocontrol bacteria therefore might depend on the respective plant species pool.

Soil abiotic and biotic properties interactively linked plant community composition to disease suppression. Root biomass increased the abundance of *Bacillus* but decreased that of *Pseudomonas* and *Actinomyces*. Root morphology differs considerably between plant species and shapes rhizosphere microbial communities^8^. In accordance, inconsistent results of root biomass effects on bacterial abundances in diverse grassland communities were recently suggested to be driven by species identity^15^. Species-specific analyses in the present study showed strong effects of the presence of *Medicago* on root biomass (F-test; p < 0.001), suggesting that a high proportion of *Medicago* roots fosters *Bacillus,* while decreasing the abundance of *Pseudomonas* and *Actinomyces*.

Further, our results suggest that root biomass also decreased the abundance of *Bacillus* via decreasing soil moisture, whereas the abundance of *Pseudomonas* and *Actinomyces* as well as soil suppression remained unaffected. Generally, soil moisture is an important driver for soil microbial communities^23^,^29^,^30^, and our results show that different microbial groups differ in their sensitivity to soil moisture and/or anaerobic micro-niches.

According to our SEM, soil pH was the most important abiotic factor increasing the abundance of *Bacillus* and decreasing pathogen suppression. The lack of effects of soil pH on the abundances of *Actinomyces* and *Pseudomonas* is not surprising since variations in pH were small (7.60–7.85) and close to the optimum of most bacterial consortia^31^. The decreasing effect of higher pH levels on pathogen suppression might have been due to a lower pH optimum of *R. solani* AG2-2 IIIB, as observed previously for *R. solani* AG3^32^.

The abundance of *Bacillus* only marginally increased pathogen suppression and differed in the response to abiotic and biotic factors in comparison to *Actinomyces* and *Pseudomonas*. This may support the observation that *Bacillus* diversity rather than abundance is involved in the suppression of *R. solani*^13^. Nevertheless, other pathogens than *R. solani* might have been affected by *Bacillus* abundance, and therefore the importance of this potential path in driving pathogen suppression should not be neglected.

A strength of our approach is to allow identifying potential causal pathways and differentiating them from spurious correlations. For instance, we showed that *Pseudomonas* abundance was not directly linked to disease suppression but their positive correlation with *Actinomyces* density could make them appear significantly linked to disease suppression in a linear regression. This suggests that soil pathogen suppression likely is not only due to the presence of certain antagonistic bacterial groups, but to facilitative interactions among bacterial groups or taxa^6^. We propose that using our SEM approach enables to evaluate the importance of soil taxa for disease suppression more precisely by enabling to differentiate effects of co-occuring microbial taxa.

Further, our two step approach fitting the SEM residuals into a linear model allowed us to reveal that a few interactions between plant species, such as *Dactylis glomerata* and *Festuca pratense*, strongly impacted pathogen suppression (Figs 3 and 4). Although we are not able at this stage to identify the underlying mechanisms explaining why some combinations of plant species specifically impact soil suppressiveness, we found potential explanations in previous studies. For instance, in presence of competitors plants increase root exudation and alter exudate composition, thereby affecting rhizosphere microbial communities^19^,^33^. In addition, different plant species are suggested to use resources in a complementary way, thereby contributing to ecosystem functioning^34^,^35^. However, whether the observed plant-plant interaction effects on pathogen suppression were due to complementary resource acquisition or plant competition driven changes in root exudation will need further evaluation.

Finally, we observed relatively low pathogen suppressiveness at diversity levels 4 (Fig. 4). Interestingly, the positive effects observed in diversity levels 2 and 8 likely were due to synergistic effects of plant species being in close proximity, i.e. were planted right next to each other (Supplementary Figure S1). In each of the 4 species treatments and in one 8 species treatment, positively interacting plant individuals were either not planted next to each other or accompanied by negatively interacting species. For instance, the species pair *Festuca-Lolium* might have hampered the positive effect of *Dactylis-Festuca* (Fig. 4). Unfortunately, due to the experimental design we were not able to directly test for 3rd order interactions. Microbial communities generally are suggested to respond with a time lag to plant community changes^36^. Our design, might have uncovered that plant-plant interaction effects on specific soil functions, such as soil suppression, are rather short-term effects. Further studies are needed to disentangle spatial and temporal effects of plant communities on the rhizosphere-environment and their implications for specific microbial functions. In addition, soil metagenomics will allow a more detailed analysis of microbial communities.

In conclusion, our results stress the importance of plant community composition as a driver of the disease suppressive potential of soils, identify important aboveground–belowground linkages and reveal complex interactions between abiotic and biotic soil properties to shape soil functions. We highlight that plant communities are involved in shaping soil-disease suppression via linkages to abiotic soil properties and the abundance of bacterial groups including species of the genera *Actinomyces, Bacillus* and *Pseudomonas*. The results represent an important step forward in understanding the complexity of pathways linking plant community composition to plant disease suppression.

## Materials and Methods

### Plants

We used a total of eight plant species, four from the two functional groups grasses and legumes that are representatives of central European mesophilic grassland Arrhenatherion communities^37^. Grasses included *Bromus erectus* Huds. (*Bromus*), *Dactylis glomerata* L. (*Dactylis*), *Festuca pratense* Huds. (*Festuca*), *Lolium perenne* L. (*Lolium*), and the legume species were *Lotus corniculatus* L. (*Lotus*), *Medicago lupulina* L. (*Medicago*), *Trifolium pratense* L. (*Trifolium p.*), and *Trifolium repens* L. (*Trifolium r*.; Appels Wilde Samen GmbH, Darmstadt, Germany). To establish plant communities of high functional diversity, we chose plant species differing considerably in their morphological, phenological and physiological traits^37^.

### Microcosm construction

Fresh soil was obtained from a bare ground area close to the field site of the Jena Experiment^37^. Prior to plantation, the soil was mixed to ensure homogeneous abiotic and biotic starting conditions, and sieved (2 mm) to remove macrofauna, roots and stones. Subsequently, 680 g of soil was mixed with 170 g 2–5 mm expanded clay; 20% of total volume (Fibo ExClay Deutschland GmbH, Lamstedt, Germany) to ensure constant humidity. The mixture was filled into PVC tubes (diameter 10 cm, height 18 cm). Upscaling the maximum of 8 plants species per 0.00785 m^2^ yields approximately 60 species per 20 m × 20 m^38^ which is the maximum diversity per area in the Jena Experiment^37^. To establish each plant species, three seeds were placed per sowing-spot; superfluous plant seedlings were removed after emergence.

### Experimental setup

Plant diversity was varied independently of functional group affiliation in a substitutive gradient ranging from one to eight species by using the random partitions design^39^. Every species was drawn at random from the species pool without replacement, such that each species was selected once at each level of diversity. Drawing was replicated three times resulting in three partitions, each containing eight plant monocultures, eight two-species mixtures, four four-species mixtures, and one eight-species mixture. One microcosm without plants per experimental block served as control (Supplementary Figure S1). We used a well-established accelerated cycle design, in which plants were harvested and the microcosms planted again with the same plant communities in a three week cycle with five cycles in total. After removal of main roots and the shoots (fine roots remained in the soil) the soil was mixed and thereafter replanted. This design allowed simulating plant succession cycles in reduced time course, and has been used before to investigate the effect of plants on the structure of bacterial communities^7^,^40^. Plant communities were grown in a climate chamber (18–22 °C; photoperiod 12 h; 150 μmol m^−2^ s^−1^ photon flux density), and watered and randomized twice a week.

### Sampling and measurements

Plant communities were destructively sampled after the completion of the fifth growth cycle. Roots of plant communities were weighed and the soil was stored at 4 °C until further use. Subsequently, total bacteria were recovered from the root systems by horizontally shaking in 20 ml cold 1/10 phosphate-buffered saline for 0.5 h (PBS)^41^. We quantified three cultivable bacterial groups covering species belonging to the genera *Actinomyces, Bacillus*, and *Pseudomonas.* These bacterial groups show a high frequency of bioactive isolates and have repeatedely been proposed as drivers of soil suppressiveness against pathogens including *Rhizoctonia solani*^6^,^13^. Diluted rhizosphere soil suspensions (2 × 10^4^–2 × 10^6^ fold) were plated on starch casein agar (SCA) containing 100 μg ml^−1^ cycloheximide^42^ to enumerate group one covering species belonging to the *Actinomyces* group. Group two covering *Bacillus* spp. was isolated by incubating the rhizosphere-soil suspension at 85 °C for 0.5 h, and plating dilutions (2 × 10^3^–2 × 10^4^ -fold) on 1/10 Tryptic Soy Agar (TSA)^43^. And group three covering pseudomonads was isolated by dilution-plating (2 × 10^4^–2 × 10^6^ fold) on 1/3 King’s B agar containing 40 μg ml^−1^ ampicillin, 13 μg ml^−1^ chloramphenicol and 100 μg ml^−1^ cycloheximide^44^,^45^ (for simplicity groups 1–3 are subsequently named as *Actinomyces, Bacillus* and *Pseudomonas*). Bacterial colonies were counted after four and additional colonies after six days (*Actinomyces*), two and three days (*Bacillus*), and three and four days (*Pseudomonas*) of growth at 20 °C. For further analyses plate counts from soil dilutions resulting in 50–500 bacterial colonies per plate were chosen.

The pH of 2 g soil was determined in a 1:10 dilution with 0.01 M CaCl_2_. The gravimetric water contend was measured by drying soil at 65 °C for three days. Thereafter, dried soil samples were ball-milled (MM 400; Retsch GmbH, Haan, Germany) for analysis of total carbon (C) and nitrogen (N) concentrations in an element analyser (Vario EL ΙΙΙ, Elementar, Hanau, Germany).

### Soil suppressiveness assay

In order to analyse the effects of previous plant community composition on pathogen suppression in the following crop, we carried out a standardized infection assay with sugar beet seedlings (*Beta vulgaris* L.; variety BELINDA, *Rhizoctonia* susceptible, KWS SAAT AG, Einbeck, Germany) and the model pathogen *Rhizoctonia solani* Kühn (AG 2–2 IIIB; IfZ, Göttingen, Germany), as described elsewhere^6^,^14^,^46^. Briefly, four Magenta boxes per experimental plot (7.7 × 7.7 × 9.7 cm; Sigma-Aldrich, St. Louis, MO, USA) were each filled with 100 g of sieved soil. One barley corn infested with *R. solani* was placed in the centre of three boxes, the fourth box without inoculum served as control. Eight sugar beet seeds (germination rate 93%) were added to each box about 0.5 cm below soil surface. The jars were incubated at 21 °C and 12 h photoperiod (photon flux density: 120 μmol m^−2^ s^−1^) and randomised every two days over a total experimental time of ten days. Dead seedlings were counted at day 2, 4, 6, and 10, and pathogen suppression was calculated as the time span until the first infection of sugar beet seedlings occurred (see statistical analyses for details).

### Statistical analyses

To estimate the disease suppressive potential of the soils after being exposed to different plant community compositions, we analysed every experimental unit separately using a monomolecular infection model^2^,^47^ describing the change of infected plants (*dI*) over time (*dt*) by an infection rate, *r*, and first infection occurrence, *t*_0_:


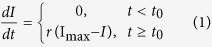


If controls were not infected by any pathogen being present in the soil, we estimated infection parameters according to the classic monomolecular model (Equation 1). Whereas, to correct for false infected controls, we fitted the monomolecular model (Equation 1) to the control data, and subsequently used the results of this fitting for parameterization of a two pathogen monomolecular infection model (Rall & Latz, *in prep.;* see Supplementary Methods for details on model fit):


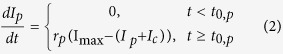



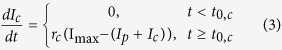


The differential equation includes two types of infected plants, plants infected by the experimentally added pathogen, *p*, and plants infected by pathogens in the control treatment, *c* (Equations 2, 3).

Subsequent analyses were performed using the statistical software R (R Core Team 2014) using the packages car^48^, lavaan^49^ and semTools^50^.

In order to disentengle linkages between plant community composition and soil suppressiveness, we used structural equation modelling (SEM), which allows the analyses of variables in a multivariate approach^51^. All variables were continuously coded. The initial model contained the exogenous variables plant diversity, presence of grasses, and presence of legumes in addition to the endogenous variables root biomass (g fresh weight; log_10_-transformed), the abiotic factors pH, total C and N content, and soil moisture (% data; logit-transformed) as well as the abundance of *Actinomyces, Bacillus* and *Pseudomonas* (colony forming units [cfu] per root system; log_10_-transformed) as potential variables explaining soil suppressiveness against *R. solani* (initial infection occurrence (*t*_*0*_); log_10_-transformed; Fig. 2a; Supplementary Table S1). This model was improved by: (i) separately analysing each endogenous variable and its dependencies in a linear regression and selecting the most parsimonious models via using the stepAICc() function^52^, respectively. Subsequently, each of those separately predefined paths were used to create a second SEM (ii) checking model modification indices for potential additional paths and undirected correlations that might not have been considered in the second model and (iii) deriving the most parsimonious model by removing non-significant pathways. Model selection was conducted by comparative fitting^53^ and using corrected Akaike’s Information Criterion (AICc)^54^,^55^ and absolute goodness of fit was determined by using χ^2^ tests (p > 0.05)^51^.

To account for additional plant effects, we performed a linear model with the residuals of the SEM fit as being dependent on the presence and 2^nd^ order interactions of the plant species. We selected the most parsimonious model via AICc. Significance of slopes were determined via t-tests.

## Additional Information

**How to cite this article**: Latz, E. *et al*. Unravelling Linkages between Plant Community Composition and the Pathogen-Suppressive Potential of Soils. *Sci. Rep.* **6**, 23584; doi: 10.1038/srep23584 (2016).

## Supplementary Material

Supplementary Information

## Figures and Tables

**Figure 1:**
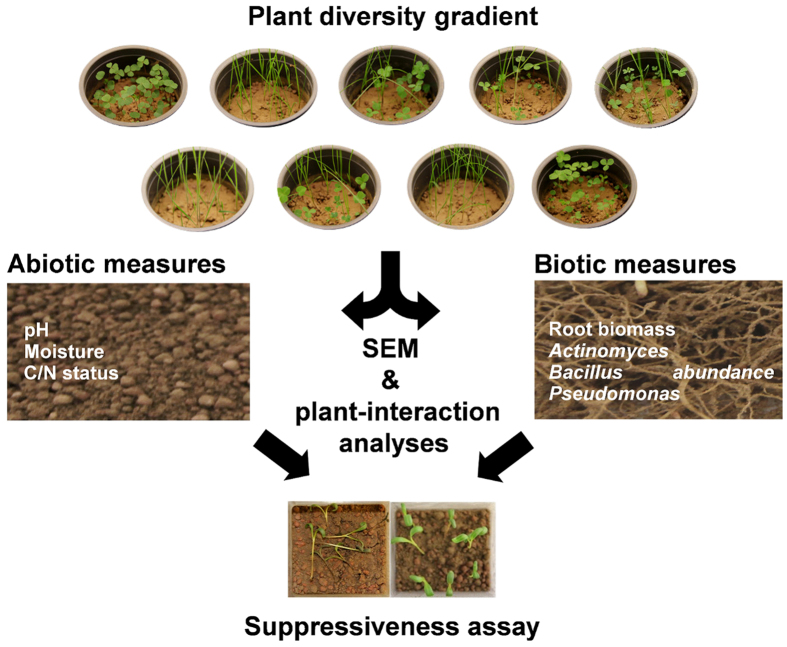
Conceptual figure. Grassland plant communities consisting of one to eight plant species were set up in a substitutive diversity gradient. To increase plant community effects on soil parameters, plant succession was simulated in growth cycles. After the fifth growth cycle, abiotic parameters were measured, plant roots were weighed, and bacterial groups including *Actinomyces, Bacillus*, and *Pseudomonas* species were enumerated. Subsequently, the soil was planted with sugar beet seedlings and infested with the model pathogen *Rhizoctonia solani*, and pathogen suppression was assessed. Pathways linking plant community composition and pathogen suppression were unravelled via structural equation modelling. In addition, plant-plant interaction effects on pathogen suppression were assessed (see methods for details).

**Table 1:**
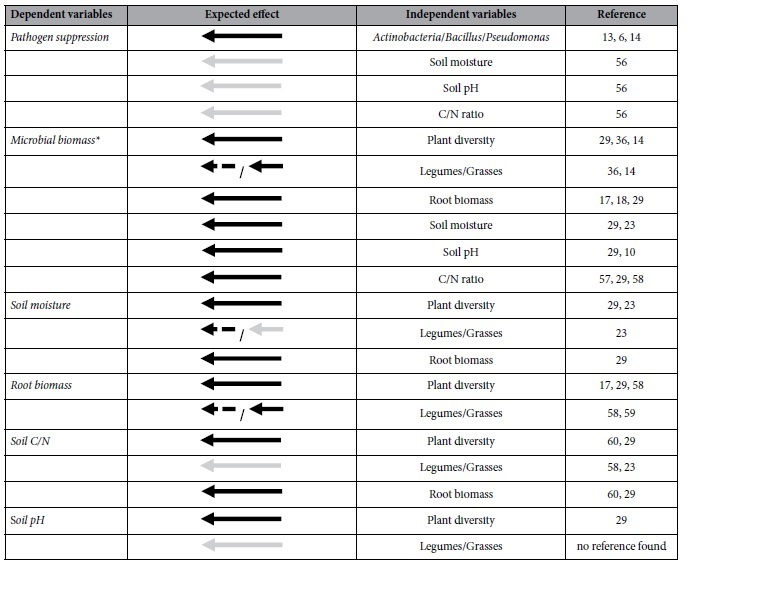
Hypotheses liable for the initial structural equation model.

See methods for details. Dashed arrows indicates negative, solid arrow indicates positive coherences. Grey arrows indicate assumed but not proofed effects or effects that gave inconsistent results. *making it likely that *Actinomyces, Bacillus, Pseudomonas*.
