# Supplementary material for: Unravelling Linkages between Plant Community Composition and the Pathogen-Suppressive Potential of Soils
Source: Sci Rep. 2016 Mar 29;6:23584. doi: 10.1038/srep23584 (PMC4810420; doi:10.1038/srep23584)
Supplement: Supplementary Information [file srep23584-s1.pdf]

Unravelling linkages between plant community composition and the pathogen-suppressive potential of soils

by E. Latz, N. Eisenhauer, B.C. Rall, S. Scheu and A. Jousset

Supplementary Material

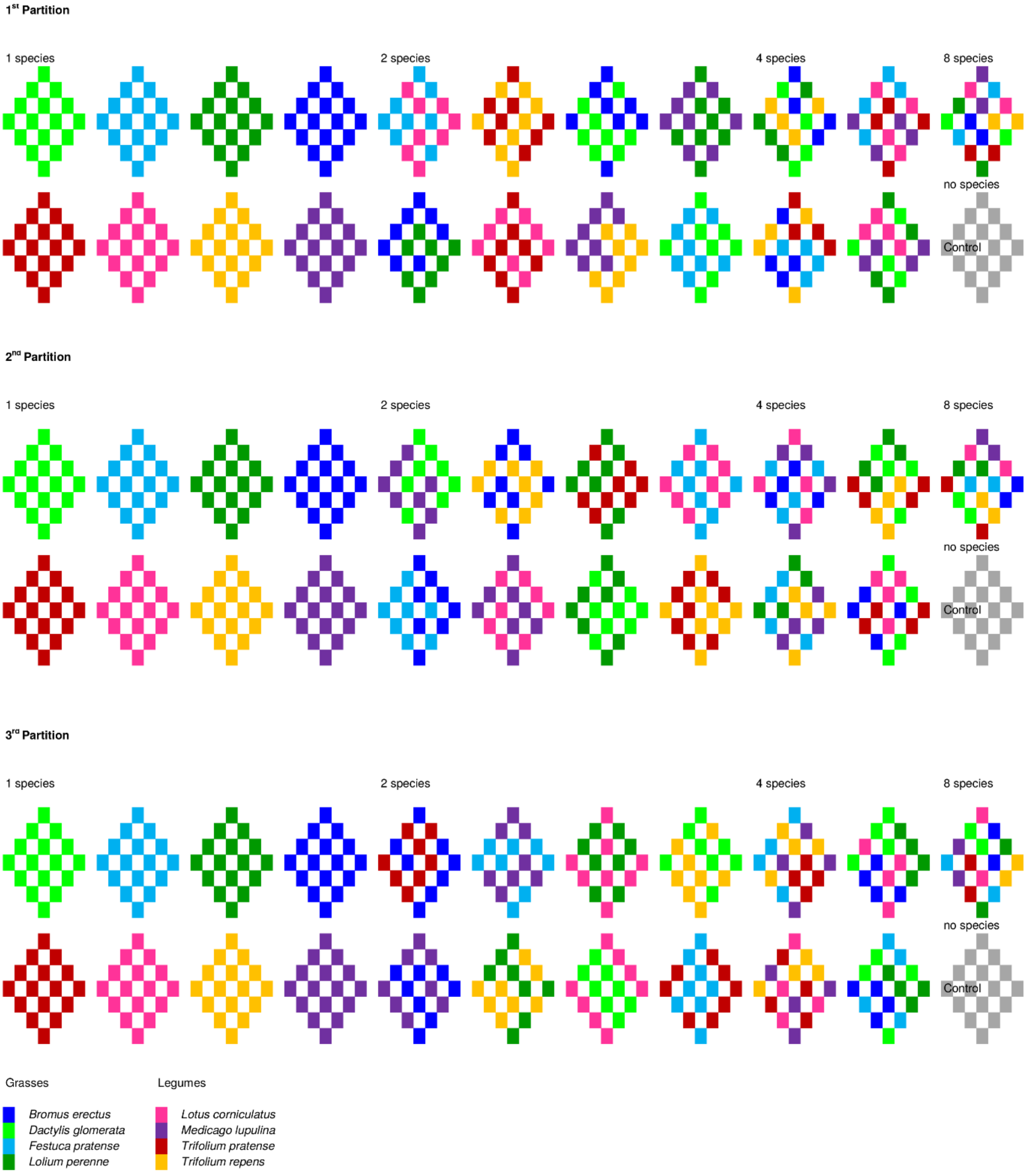

Supplementary Figure 1. Experimental setup of the substitutive plant species richness gradient. Plant diversity was varied independently of functional group affiliation by using the random partitions design (Bell et al. 2009). Displayed is the spatial arrangement of the last plant growth cycle (fifth). Each small square represents one plant individual; each main square represents one plant community.

Supplementary Table 1. Results of the final structural equation model (X^2_{21} = 17.22; P-value = 0.698), showing effects of direct and indirect (through changes in soil pH, root biomass, soil moisture and bacterial abundances) effects of plant community composition on pathogen suppression.

			Estimate	S.E.	C.R.	
**Regressions**						
Soil pH	←	Plant diversity	0.077	0.05	1.66	.
Soil moisture	←	Root biomass	-0.207	0.03	-6.19	***
Root biomass	←	Plant diversity	0.478	0.20	2.41	*
	←	Grass presence	-1.333	0.74	-1.79	.
Bacillus	←	Soil pH	0.645	0.22	3.00	**
	←	Soil moisture	0.443	0.19	2.28	*
	←	Root biomass	0.308	0.07	4.73	***
	←	Grass presence	0.797	0.30	2.66	**
Pseudomonas	←	Root biomass	-0.057	0.01	-5.26	***
	←	Grass presence	-0.272	0.07	-4.05	***
	←	Legume presence	0.192	0.07	2.83	**
Actinomyces	←	Root biomass	-0.047	0.02	-3.02	**
	←	Grass presence	-0.323	0.10	-3.33	**
	←	Legume presence	0.529	0.10	5.38	***
Pathogen suppression	←	Plant diversity	0.149	0.04	3.74	***
	←	Legume presence	-0.442	0.18	-2.47	*
	←	Soil pH	-0.291	0.10	-2.86	**
	←	*Pseudomonas*	-0.428	0.27	-1.60	
	←	*Actinomyces*	0.547	0.20	2.71	**
	←	*Bacillus*	0.057	0.05	1.15	
**Covariances**						
Actinomyces	↔	*Pseudomonas*	0.000	0.00	3.00	**
Plant diversity	↔	Grass presence	0.002	0.00	2.32	*
	↔	Legume presence	0.003	0.00	2.46	*
Grass presence	↔	Legume presence	-0.001	0.00	-2.82	**

Given are non standardized path coefficients (estimates), standard error of regression weight (S.E.) and the critical value for regression weight (C.R.; z = estimate/ S.E.). Asterisks denote the level of significance: . P ≤ 0.1; *P ≤ 0.05; **P ≤ 0.01; ***P < 0.001. For more information of exogenous and endogenous variables as well as on model fit see main text.

Supplementary Table 2. Effects of plant community composition on pathogen suppression as indicated by additional, sequential analyses (see text for details).

	**Number of grasses**			***Bromus erectus***			***Dactylis glomerata***			***Festuca pratense***			***Lolium perenne***		
1. step	Estimate	S.E.	t-value	Estimate	S.E.	t-value	Estimate	S.E.	t-value	Estimate	S.E.	t-value	Estimate	S.E.	t-value
Intercept	0.578	0.010	58.74 ***	0.591	0.008	71.99 ***	0.589	0.008	71.82 ***	0.591	0.008	71.13 ***	0.589	0.008	72.54 ***
Plant number/ identity	0.016	0.006	2.50 *	0.018	0.016	1.17	0.026	0.015	1.75 .	0.018	0.015	1.16	0.025	0.015	1.64
DF/R^2	59/0.10			59/0.02			59/0.05			59/0.02			59/0.04		
2. step															
Intercept	-0.005	0.114	-0.47	-0.018	0.011	-1.58	-0.016	0.011	-1.39	-0.018	0.011	-1.60	-0.016	0.011	-1.41
Plant diversity	0.002	0.004	0.58	0.008	0.004	1.96 .	0.007	0.004	1.73 .	0.008	0.004	1.98 .	0.007	0.004	1.74 .
DF/R^2	59/0.01			59/0.06			59/0.05			59/0.06			59/0.05		
	**Number of Legumes**			***Lotus corniculatus***			***Medicago lupulina***			***Trifolium pratense***			***Trifolium repens***		
1. step	Estimate	S.E.	t-value	Estimate	S.E.	t-value	Estimate	S.E.	t-value	Estimate	S.E.	t-value	Estimate	S.E.	t-value
Intercept	-0.016	0.011	-1.41	0.593	0.008	70.87 ***	0.588	0.008	71.88 ***	0.595	0.008	71.67 ***	0.594	0.008	70.85 ***
Plant number/ identity	0.007	0.004	1.74 .	0.011	0.015	0.70	0.027	0.015	1.79 .	0.005	0.016	0.33	0.009	0.015	0.61
DF/R^2	59/0.05			59/0.01			59/0.05			59/0.00			59/0.01		
2. step															
Intercept	-0.013	0.012	-1.08	-0.020	0.011	-1.77 .	-0.015	0.011	-1.38	-0.022	0.011	-1.91 .	-0.021	0.011	-1.81 .
Plant diversity	0.005	0.004	1.45	0.009	0.004	2.20 *	0.007	0.004	1.71 .	0.009	0.004	2.36 *	0.009	0.004	2.24 *
DF/R^2	59/0.03			59/0.08			59/0.05			60/0.06			59/0.08		

The tables were generated with the R-function summary(), showing the probability of a variable to be zero (two-tailed t-test). Asterisks denote the level of significance: . $P \leq 0.1$; *$P \leq 0.05$; **$P \leq 0.01$; ***$P < 0.001$. DF, degrees of freedom; R^2 coefficient of determination.

Supplementary Methods. The models were fitted to the data by using a numerical integration routine (lsoda() from the **deSolve** package in R [Soetaert *et al.* 2010; R Core Team 2014]) combined with the maximum likelihood optimizer mle2() from the package **bbmle** (Bolker & R Development Core Team 2014). To ensure the estimation of high infection rates, we chose a step size for the numerical integration routine (lsoda()) of 0.025 (default is 0.1) and allowed the maximum likelihood optimizer (mle2()) to try at maximum 10,000 iteration steps (control = list(maxit=10000); default is 100). Integrative maximum likelihood estimations of non-linear models may result in local optima that lead to biased results or, in dependence of the starting parameters, fail completely (Bolker 2008). To cope with this problem, we repeated each analyses 100 times with starting parameters randomly sampled out of a uniform distribution with: $0.0001 \leq r \leq 3$; $0.0001 \leq t_0 \leq 0.8 \times \min(t_{Infectedcontrol>0})$; $0.01 \leq r_p \leq 15$; $0.001 \leq t_{0p} \leq 0.8 \times \min(t_{Infectedtreatment>0})$.

We checked for the quality of the fitted parameters in each experimental unit by (1) selecting all model fits out of the hundred fittings where the ΔAIC was below 2, and (2) we calculated the coefficient of variation for t_0 of the remaining model fits. Only if the coefficient of variation was below 0.05, we rated the fit as trustful. Subsequently, we chose the value for t_0 of the fit with the lowest AIC for further analyses.

Bolker, B. & R Development Core Team. (2014) *Bbmle: Tools for General Maximum Likelihood Estimation. R Package Version 1.0.17*.

Soetaert, K., Petzoldt, T. & Setzer, R.W. (2010) Solving differential equations in R: package deSolve. *Journal of Statistical Software*, **33**, 1–25.
